# I "Gut" Rhythm: the microbiota as a modulator of the stress response and circadian rhythms

**DOI:** 10.1111/febs.17400

**Published:** 2025-01-22

**Authors:** Gabriel S. S. Tofani, Gerard Clarke, John F. Cryan

**Affiliations:** ^1^ APC Microbiome University College Cork Ireland; ^2^ Department of Anatomy & Neuroscience University College Cork Ireland; ^3^ Department of Psychiatry & Neurobehavioural Science University College Cork Ireland

**Keywords:** circadian rhythm, gut microbiota, microbiota‐gut–brain axis, stress response

## Abstract

Modern habits are becoming more and more disruptive to health. As our days are often filled with circadian disruption and stress exposures, we need to understand how our responses to these external stimuli are shaped and how their mediators can be targeted to promote health. A growing body of research demonstrates the role of the gut microbiota in influencing brain function and behavior. The stress response and circadian rhythms, which are essential to maintaining appropriate responses to the environment, are known to be impacted by the gut microbiota. Gut microbes have been shown to alter the host's response to stress and modulate circadian rhythmicity. Although studies demonstrated strong links between the gut microbiota, circadian rhythms and the stress response, such studies were conducted in an independent manner not conducive to understanding the interface between these factors. Due to the interconnected nature of the stress response and circadian rhythms, in this review we explore how the gut microbiota may play a role in regulating the integration of stress and circadian signals in mammals and the consequences for brain health and disease.

## Graphical abstract

The stress response and circadian rhythms are essential to maintain appropriate responses to the environment and are known to be impacted by the gut microbiota. Due to the interconnected nature of these important components of physiology, in this review we explore how the gut microbiota may play a role in regulating the integration of stress and circadian signals in mammals and the consequences for brain health and disease.

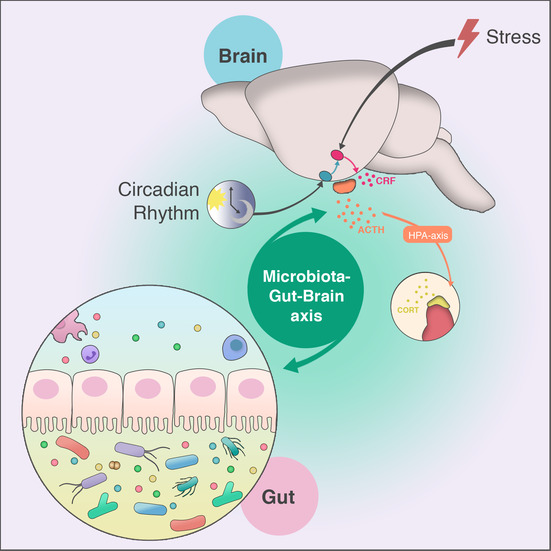

**Abbreviations**
ACTH: adrenocorticotropic hormone
AD: Alzheimer's disease
AII: angiotensin II
ANS: autonomic nervous system
AVP: arginine vasopressin
BBB: blood–brain barrier
CALB: calbindin
CALR: calretinin
CNS: central nervous system
CRF: corticotropin‐releasing factor
FMT: fecal microbiota transplant
GF: germ free
GR: glucocorticoid receptor
GRP: gastrin‐releasing peptide
HD: Huntington's disease
HDAC3: histone deacetylase 3
HFD: high‐fat diet
HPA: hypothalamic–pituitary–adrenal
ILC3: innate lymphoid cell
LPS: lipopolysaccharide
MC2R: melanocortin 2 receptor
MDD: major depressive disorder
mENK: enkephalin
MR: mineralocorticoid receptor
NAc: nucleus accumbens
NMS: neuromedin S
NT: neurotrophin
PD: Parkinson's disease
PFC: prefrontal cortex
PNS: parasympathetic nervous system
PTSD: post‐traumatic stress disorder
PVN: paraventricular nucleus
SAM: sympathomedullary
SCFA: short‐chain fatty acid
SCN: suprachiasmtic nucleus
SNS: sympathetic nervous system
TLR: Toll‐like receptor
VIP: vasoactive intestinal peptide
VTA: ventral tegmental area

## Introduction

The stress response and circadian rhythms are interconnected to mantain homeostasis and appropriate responses to the environment. Due to a growing number of research studies demonstrating that the gut microbiota can regulate these two components of physiology, we review the current literature indicating how gut microbes could play a role in the interaction between the stress response and circadian rhythms in mammals. First, we define the microbiota–gut–brain axis and the pathways through which the microbiota can alter brain physiology. Next, we explore the stress response and circadian rhythms, and how they can be shaped independently by the microbiota. Lastly, we discuss the evidence supporting the role for gut microbes as a key interface between circadian and stress biology.

## Microbiota–gut–brain axis

The gut microbiota is composed of trillions of microorganisms, which include bacteria, archaea, eukaryotes, and viruses, that reside in the gastrointestinal tract [1]. These microorganisms co‐evolved with their hosts resulting in an intertwined relationship between the gut microbiota and host physiology [2]. Such interaction is important for an organism's survival as the microbiota plays a key role in nutrition [3], immune development, and pathogen defense [4].

The microbiota–gut–brain axis refers to the bidirectional communication between the gut microbiota and the brain [5]. This field has gained traction over the last few years as more and more clinical and preclinical studies demonstrate the impact of the gut microbiota on host brain function with implications that range from behavior to disease severity and progression [6, 7, 8, 9]. The importance of this field of study is exemplified by the fact that alterations to gut–brain communication are observed in many psychiatric and neurological disorders [10, 11, 12, 13]. Understanding how the gut microbiota can shape brain physiology can provide us with novel therapeutic tools to target the microbiota to improve health and quality of life.

### Pathways of communication

**Fig. 1:**
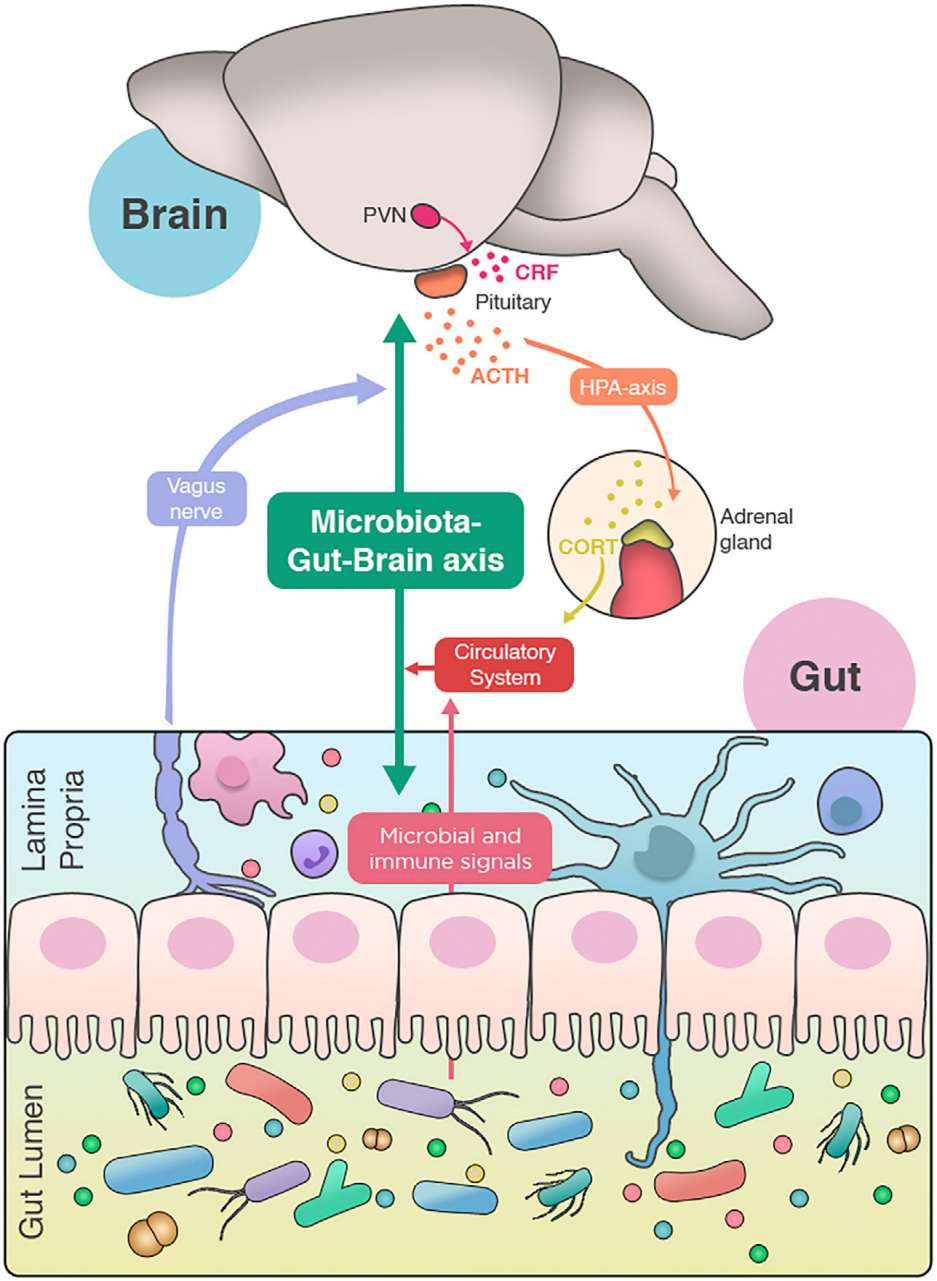
Pathways of communication of the microbiota–gut–brain axis. The gut microbiota has been shown to modulate brain physiology through different pathways which include: HPA axis, vagus nerve, immune system, and microbial metabolites. ACTH, adrenocorticotropic hormone; CORT, glucocorticoid; CRF, corticotropin‐releasing factor; HPA axis, hypothalamic–pituitary–adrenal axis; PVN, paraventricular nucleus of the hypothalamus.

Communication between the gut microbiota and the central nervous system (CNS) involves different direct or indirect pathways, such as the autonomic nervous system (ANS), endocrine system, immune system, and microbial metabolites [5, 7, 14] (Fig. 1). Although a lot of progress has been made in identifying such pathways, more work is needed to identify the exact mechanisms underlying gut–brain communication. More importantly, due to the overlapping nature of such pathways, it is key to understand how these different signals are integrated to shape brain function and behavior.

#### Hypothalamic–pituitary–adrenal axis

The hypothalamic–pituitary–adrenal (HPA) axis is often highlighted as being one of the main pathways of communication between the gut microbiota and the brain [15, 16, 17]. Glucocorticoid hormones are the main output of the HPA axis and they have widespread effects throughout the body, representing a major stress and circadian signal key in maintaining appropriate responses to the environment [18]. Microbial status can influence the circulating levels of glucocorticoids both at baseline and following stress [16, 19, 20]. Moreover, due to their role in modulating neuronal function [21], changes/fluctuations in circulating glucocorticoids have been associated with alterations in sociability, and anxiety and depression‐like behavior, behavioral features also associated with changes in gut microbiota composition and/or function [22, 23]. Lastly, it is important to note that not only can microbial status alter glucocorticoid concentrations, but these hormones can also reciprocally influence gut microbiota composition [24, 25].

#### Vagus nerve

As part of the ANS, the vagus nerve is the fastest and most direct way for the gut microbiota to relay signals to the brain [26]. This nerve is the 10th cranial nerve and has an important role in maintaining homeostasis by transmitting information from the brain to the viscera and vice versa [27]. This rapid communication between gut and brain is the subset of enteroendocrine cells in the gut that are able to synapse with the vagus to transmit information from the gut lumen to the brainstem [28]. Evidence that the vagus may underlie some of the effects of the gut microbiota on the brain has emerged as studies have shown that some of the positive effects of probiotics on behavior are vagus dependent [13, 29, 30].

#### Immune system

The immune system is closely intertwined with the gut microbiota, as immune cells residing in the gut can shape microbial composition and microbial signals can alter immune function [4]. Although most studies of microbiota‐immune interactions have focused on the periphery, studies have also demonstrated that gut microbes can lead to alterations in immune cells in the brain. Microglia in GF mice display an immature phenotype [31] and disruptions to the early‐life microbiota can lead to alterations in the morphology of these immune cells that are coupled with behavioral changes [32]. Moreover, microbial metabolites have been shown to modulate astrocyte activity and inflammation in the CNS [33]. These findings have led to the hypothesis that immune changes in the context of gut–brain communication may underlie some of the changes in brain function driven by the microbiota [34].

#### Microbial metabolites

Some microbial metabolites have also surfaced as important drivers of gut microbiota–brain communication. Short‐chain fatty acids (SCFA) are microbial metabolites originating from the fermentation of dietary fibers [35]. Research suggests that such metabolites might be able to cross the blood–brain barrier (BBB) [36, 37] and receptors for SCFAs are expressed across nervous tissues both centrally and in the periphery [38]. Moreover, these metabolites can lead to vagus nerve activation and modulate immune and endocrine signals [39].

Tryptophan, an essential amino acid, can be metabolized by the microbiota and converted into indole compounds [40]. These indoles can activate aryl hydrocarbon receptors (AHR) which in turn modulate the host immune system [41]. Additionally, these metabolites can also interact with nervous tissues, promoting nerve regeneration [42]. Lastly, tryptophan can be converted into serotonin, a key neurotransmitter for both gut and brain function, highlighting its role in gut–brain communication [40]. Although some bacteria are capable of producing serotonin, there is also evidence for indirect regulation of this host metabolic pathway by gut microbes and the metabolites they produce [40].

## Stress response

**Fig. 2:**
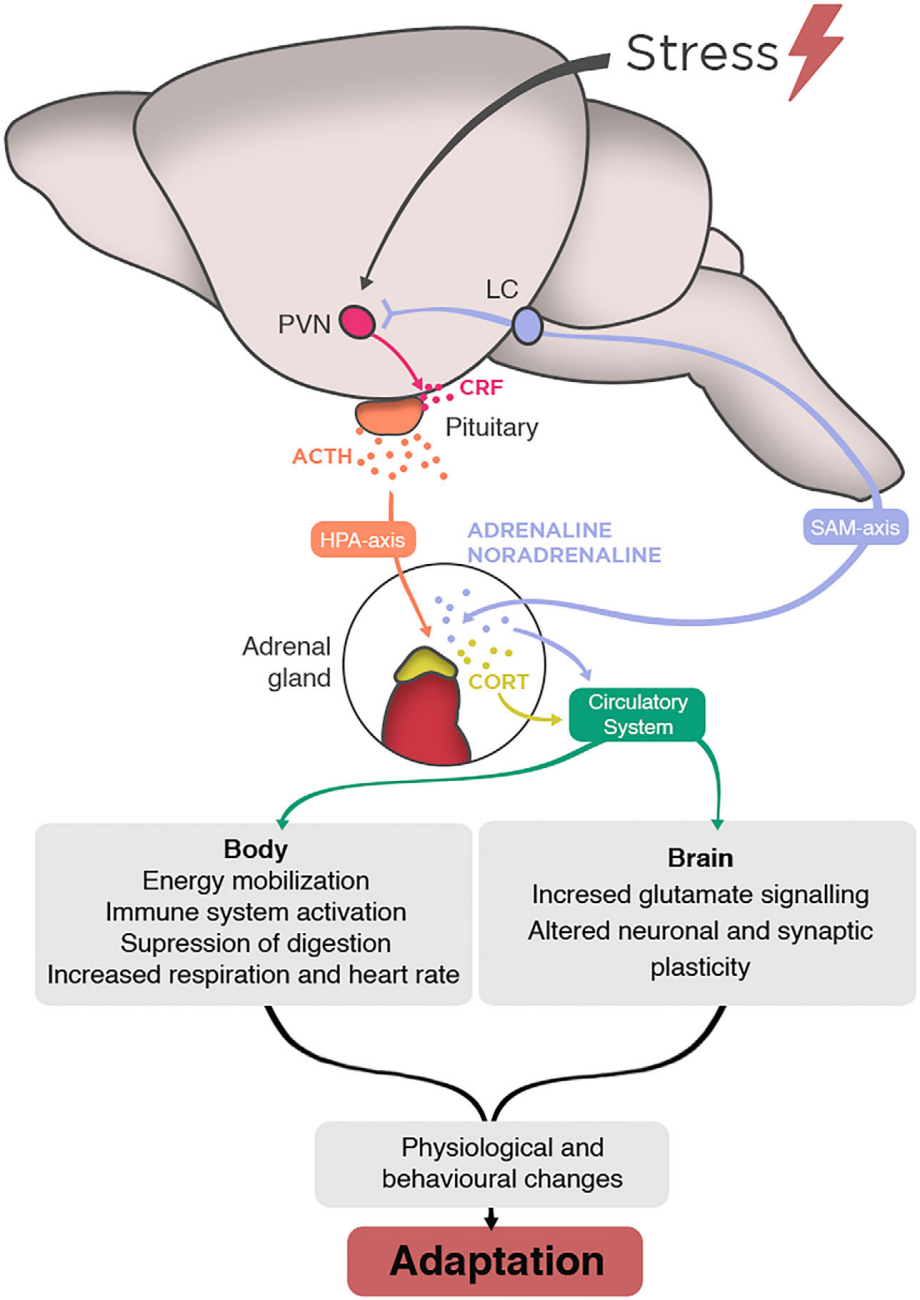
Overview of the stress response. Stressful stimuli lead to activation of the PVN in the hypothalamus which will lead to subsequent activation of the locus coeruleus and the SAM axis, leading to rapid release of adrenaline and noradrenaline. In parallel, activation of the PVN will also generate the release of CRF which leads to the activation of the HPA axis, ultimately resulting in an increase of glucocorticoids in circulation. Moreover, when activated, the stress system generates changes in physiological processes in the brain and body which in turn results in adaptation to the stressor. CORT, glucocorticoid; CRF, corticotropin‐releasing hormone; HPA axis, hypothalamic–pituitary–adrenal axis; LC, locus coeruleus; PVN, paraventricular nucleus of the hypothalamus; SAM axis, sympathomedullary axis.

The stress response is an integral part of how living organisms adapt to changes in the dynamic environment around them. Although no clear definition of stress has been agreed upon, stress can be broadly defined as an intrinsic or extrinsic stimulus that leads to an actual or perceived disturbance of homeostasis generating a subsequent biological compensatory response, known as the stress response [43, 44]. The stress response is composed of a complex set of physiological and behavioral responses that allow organisms to adapt to environmental challenges [45] (Fig. 2). When the stress response is inadequate or exacerbated, it can lead to alterations in brain, immune, gastrointestinal, cardiovascular, and metabolic function [44].

To understand the importance of the stress response in maintaining health, we must explore the biological mediators of such responses, of which the two main actors are the HPA axis and the ANS containing both central and peripheral components [43]. These mediators will respond differently, generating adaptive or maladaptive responses that depend on not only the duration, frequency, and time‐of‐day of the stressor, but also stage of life and genetics of the individual [46, 47, 48, 49]. Moreover, the HPA axis and ANS are highly interconnected with different brain regions that allow the organism to respond adaptively to different kinds of stressors [50].

### Hypothalamic–pituitary–adrenal axis

When faced with a stimulus that is perceived to be threatening, different neural circuits are recruited and ultimately the corticotropin‐releasing factor (CRF) neurons in the paraventricular nucleus of the hypothalamus (PVN) are activated leading to further activation of the HPA axis, which constitutes one of the pillars of the stress response [45]. PVN neurons also synthesize two other neuropeptides that have roles in regulating the stress response: arginine vasopressin (AVP) and oxytocin [51]. Once these neurons are activated, CRF is released from the PVN into fenestrated portal capillaries by nerve endings and then travels to the anterior pituitary gland where it activates corticotropic cells to release adrenocorticotropic hormone (ACTH) into circulation, where it reaches and activates melanocortin type 2 receptors (MC2R) in the adrenal cortex and stimulates the synthesis and later release of glucocorticoids in the circulation [52]. The main glucocorticoid in rodents is corticosterone and its equivalent in humans is cortisol [53].

Once in circulation, glucocorticoids can reach almost every cell in the body and cross the cell membrane to bind to glucocorticoid (GR) and mineralocorticoid (MR) receptors [45]. Nonactivated GR and MR reside in the cytoplasm and upon activation, these receptors translocate to the nucleus where they bind glucocorticoid‐response elements located in the promoter region of different target genes, regulating their expression positively or negatively [54]. Moreover, glucocorticoids are involved in regulating HPA axis activation, creating a negative feedback loop capable of halting the stress response [55]. While MRs are sensitive to low concentrations of glucocorticoids, GRs are sensitive to both basal and stress‐induced glucocorticoid concentrations, and when activated in the PVN and pituitary, act as a negative regulator, leading to the reduction in the release of CRF and ACTH, respectively [45].

### Autonomic nervous system

Besides the HPA axis, stress also leads to the activation of the ANS, which is comprised of the sympathetic nervous system (SNS), the sympathomedullary (SAM) system, and the parasympathetic nervous system (PNS). In particular, the activation of the SNS results in a rapid response to stressors leading to the release of noradrenaline centrally, and activation of the SAM system leads to release of noradrenaline and adrenaline into circulation by the adrenal medulla [46]. Together, these two neurotransmitters further regulate the cardiovascular, respiratory, gastrointestinal, endocrine, and other systems in order to allow the organism to cope with the stressor [45].

### Physiological impacts of stress on brain and behavior

Stress is known to shape brain development, function, and behavior [56]. This modulation is often observed as changes in memory, cognition, social behavior, and coupled with structural changes in the brain [44, 56, 57]. Moreover, stress is intrinsically linked to mental health, with stress exposure and dysfunction of the stress response is associated with the development and severity of conditions such as anxiety and depression [58, 59, 60]. Although there is a well‐established effect of stress on the brain, the consequences of such effects depend on the type, timing, and duration of the stressor.

#### Acute stress

To better understand the mechanisms through which stress can remodel brain function, we first need to explore how the brain responds to acute stress. Acute stress is a key component of chronic stress and the acute responses can sometimes be (mal)adaptive or even lead to mental health disorders [61]. In particular, cognitive function and memory are known to be affected by acute stress [62, 63]. In humans, these effects can be either positive or negative depending on the duration, severity, and type of stress exposure, characteristics of the individual, and the behavioral/cognitive domain under evaluation [62, 64]. In rodents, impairments in special memory and object recognition have been observed following acute stress [63, 65]. These behavioral alterations are linked to stress signaling pathways, with firing patterns in the hippocampus responding differently depending on the concentration of glucocorticoids [66]. Moreover, glucocorticoids and CRF have also been shown to regulate memory consolidation and synaptic plasticity through signaling in the basolateral amygdala [67, 68].

Acute stress is also able to shape social behavior, but such modulation seems to be variable in the literature, with some studies showing increased or decreased sociability depending on the type of stress and test used. In rodents, some studies have shown that acute stress increases social bonding and reduces aggression [69, 70], while others indicate an increase in non‐social and aggressive behaviors [71, 72]. Data in humans indicate that acute stress can lead to increased prosocial behavior, which can act as a stress‐buffering strategy [73, 74]. Together, these data demonstrate that more studies are needed to better understand the factors shaping alterations in social behavior following stress. Factors such as the relative duration, intensity, and valence of the stress will play a key role coupled with sex and genetic effects [75]. Lastly, the effects of acute stress on anxiety have also been identified. Acute stress leads to an increase in anxiety‐like behavior, which is mediated by glucocorticoids and the noradrenergic system [76, 77].

#### Chronic stress

Similar to acute stress, chronic stress exposure can alter brain function and behavior [56]. In humans, chronic stress can lead to long‐lasting alterations in stress responsivity and is associated with the development of stress‐related disorders [78, 79]. Repeated stress exposure in rodents has been shown to lead to morphological changes in brain structure, with hippocampal neurons developing atrophy of apical dendrites and reduced neurogenesis [80, 81]. In parallel, chronic stress leads to changes in hippocampal‐dependent behavior such as cognition and memory [82, 83]. Moreover, decreased hippocampal volume was observed in individuals with PTSD when compared to controls and are associated with short‐term memory deficits [84].

Social behavior has also been shown to be affected by chronic stress, where social interactions are reduced in different sociability tests [69, 85, 86]. Additionally, chronic social defeat stress leads to increased social avoidance to an unknown conspecific, which has been attributed to glucocorticoid signaling in the brain [87, 88]. In the context of anxiety, chronic stress has been observed to increase anxiety‐like behavior [89, 90]. Moreover, chronic unpredictable mild stress is often used as a model of depression [91].

### Gut microbiota as a modulator of the stress response

The intertwined relationship between stress and the gut microbiota is well established [92]. Stress is known to alter gut microbiota composition/function, with supporting data coming from many preclinical [93, 94, 95] and clinical studies [96, 97]. Parallel to changes in composition, the microbiota responds to both chronic and acute stress by changing its metabolic output and modulating indices of gut barrier integrity and function [75]. Moreover, gut microbes are an important factor in shaping the response to stress in both human and animal models [19, 98].

#### Regulation of stress mediators

Although the effects of the microbiota on stress are well studied, the mechanisms underlying such modulation are still being explored. One of the ways that the gut microbiota can shape the host's response to stress is by modulating the levels of circulating glucocorticoids and catecholamines. GF mice have exacerbated levels of glucocorticoids and ACTH following stress, which can be reversed upon host colonization [16]. Glucocorticoid secretion is not only regulated by the gut microbiota but is also capable of shaping microbial composition [24, 25]. Moreover, GF mice display elevated circulating levels of adrenaline and noradrenaline, which are partially recovered following colonization [99]. Additionally, ligands derived from the microbiota have been shown to modulate the storage and secretion of catecholamines in the adrenals [100]. The microbiota also has an important role of shaping HPA axis development which can lead to alterations in stress responsivity later in life. Early‐life stress can alter the corticosterone stress response in adulthood, which correlates with changes in the gut microbiota [93, 101]. Lastly, prenatal stress can also lead to alterations in the microbiota in adulthood, which is accompanied with higher plasma corticosterone after a stressful social interaction [102].

#### Stress‐sensitive behaviors

This regulation of stress signaling pathways by the microbiota can ultimately translate to alterations in stress‐sensitive behaviors. The alterations in the neuroendocrine response to stress in GF mice are accompanied with enhanced anxiety‐like behavior [103]. Antibiotic exposure can attenuate some of the effects of chronic stress on anxiety and depressive‐like behavior [104]. The modulation of social behavior by the gut microbiota is also directly linked to stress responsivity, where both antibiotic‐treated and GF mice show reduced social behavior, increased neuronal activation in the PVN, and increased plasma corticosterone following a social interaction [23]. Moreover, transplantation of fecal microbiota from depressed patients to GF mice leads to increased depressive‐like behavior which is associated with higher levels of corticosterone, ACTH and CRF [105]. Similarly, transfer of the microbiota from mice that underwent chronic stress led to increased anxiety and depression‐like behavior [106, 107]. The gut virome, a relatively underappreciated component of the gut microbiome [108], has also been implicated in mediating the effects of stress, with an autochthonous virome transfer being able to protect against the alterations induced by stress in social and anxiety‐related behaviors [109].

Interestingly, manipulation of the gut microbiota through dietary interventions can also shape behavior outcomes associated with stress. Administration of probiotics such as *Lactobacillus helveticus* and *Akkermansia muciniphila* have been associated with a reduction on depressive‐like behaviors following chronic stress exposure [110, 111, 112]. Prebiotics, such as inulin and sialyllactose, have also been found to ameliorate some of the behavioral effects of chronic stress [95, 113]. Moreover, administration of a psychobiotic diet can lead to reduced levels of perceived stress in humans [98].

#### Gut microbiota and stress‐related disorders

Alterations in gut microbial composition have been observed in many stress‐related disorders such as depression, anxiety, and PTSD, which further indicate the interaction between stress and the gut microbiota [12]. In the context of depression, a study has found an increase in overall microbial richness and alterations to the three most abundant phyla (Firmicutes, Bacteroidetes, and Proteobacteria) in patients with active‐MDD when compared to healthy controls [114]. Moreover, the neuroactive potential of the microbiota has also been reported to be altered in depression [115]. A systematic review of the effects of probiotics in depression indicated that the administration of probiotics can significantly decrease depression scores [116]. As for anxiety disorders, a study characterized the microbiota of patients with generalized anxiety disorder and found reduced microbial richness and SCFA‐producing bacteria [117]. Lastly, individuals with post‐traumatic stress disorder (PTSD) display lower microbial diversity [118], and some phyla (Actinobacteria, Lentisphaerae, and Verrucomicrobia) have been found to be associated higher PTSD scale score [119]. Although a lot of studies report changes in microbial composition in stress‐related disorders, such alterations are not always consistent. Meta‐analysis exploring this issue reports that the most consistent changes are related to a decrease in anti‐inflammatory butyrate‐producing bacteria [12, 120, 121]. These alterations in gut microbial composition in stress‐related conditions, together with the preclinical data available, demonstrate the need to understand the mechanisms through which the gut microbes modulate stress responsiveness. A better understanding of the relationship between stress and the gut microbiota will then allow for the development of precision microbiota‐based strategies to improve the quality of life of people suffering from stress‐related disorders.

## Circadian rhythm

Circadian rhythms are evolutionarily conserved adaptations to the environment that are present in almost all living organisms and are suggested to have first appeared in some form over 2.5 billion years ago [122, 123]. In animals, circadian regulation is key to maintain homeostasis and adequate responses to the environment, coordinating a range of metabolic [124], endocrine [125], immune [126], and behavioral processes [127]. The importance of endogenous time‐keeping is even more evident when we consider the diverse spectrum of diseases that contain a circadian component ranges from neurodegenerative diseases [128, 129] and psychiatric disorders [130], to obesity and metabolic syndrome [131].

**Fig. 3:**
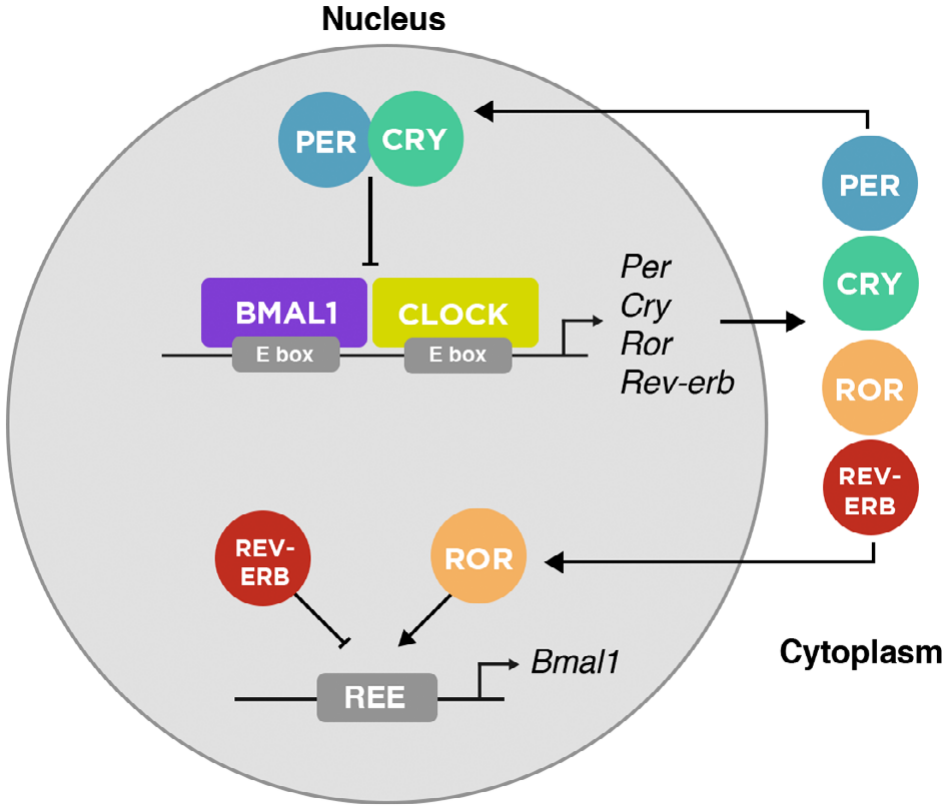
Overview of the mammalian molecular circadian clock. The molecular clock is comprised of a core loop where the heterodimer of BMAL1 and CLOCK activates the expression of Per, Cry, Ror, and Rev‐Erb. Once translated into proteins in the cytoplasm, PER and CRY translocate to the nucleus where they inhibit the transcriptional activity of BMAL1 and CLOCK. In parallel, ROR and REV‐ERB regulate Bmal1 expression positively and negatively, respectively.

The endogenous circadian processes happen at the cellular level, with a cell‐autonomous transcription‐translation feedback loop that lasts approximately 24 h [132]. In mammals, the ‘core’ loop is composed of two heterodimeric transcription factors CLOCK (Clock Circadian Regulator) and BMAL1 (Basic Helix–Loop–Helix ARNT Like 1), that activate the transcription of *Per1/Per2* (Period) and *Cry1/Cry2* (Cryptochromes) that once translated into proteins (PER and CRY) act as repressors [132, 133]. Another important regulating loop is composed of RORα/β (RAR Related Orphan Receptor A/B) and REV‐ERBα/β (Nuclear Receptor Subfamily 1 Group D) proteins that are also activated by BMAL1/CLOCK, and compete for binding sites on the BMAL1 gene, subsequently acting as positive (ROR) and negative (REV‐ERB) regulators [133, 134] (Fig. 3). This interconnected feedback loop system generates a refined control of circadian function and is key to maintaining homeostasis [135, 136].

### Central circadian rhythmicity and regulation

The brain plays a central role in regulating circadian rhythms and more importantly the synchronization of different tissues across the body. Studies in the 70's headed by Robert Y. Moore and Irving Zucker first identified the suprachiasmatic nucleus (SCN) as a major circadian component of the brain. Their work demonstrated that lesions to this region led to disruption of the body's circadian rhythm in endocrine [137] and behavioral functions [138]. Today this region is recognized as the master pacemaker for its role in synchronizing clocks in other brain regions and the periphery [139].

The SCN is located in the hypothalamus, being a paired neuronal structure on either side of the third ventricle [140]. Around 20 000 neurons compose different populations in the SCN and based on their peptide phenotypes and afferent projections, two subdivisions have been described: the ‘core’ and the ‘shell’ [141, 142]. The ventral core region sits on top of the optic chiasm and receives input from the retina; it is composed of vasoactive intestinal polypeptide (VIP), gastrin‐releasing peptide (GRP), GABA, calbindin (CALB), calretinin (CALR), neuromedin S (NMS), and neurotensin (NT) producing neurons. On the other hand, the more dorsal shell region receives inputs from the core region it partially encapsulates and is composed mainly of arginine vasopressin (AVP), GABA, CALB, angiotensin (AII), met‐enkephalin (mENK), and NMS‐positive neurons [143, 144].

One of the major circadian environmental cues to the SCN comes in the form of light. Light input via the retina travels through the retino‐hypothalamic tract, which is composed of axons of photosensitive retinal ganglion cells [145], and results in the release of glutamate and pituitary adenylate cyclase‐activating polypeptide at the synapse that activate SCN neurons of the core region, leading to direct entrainment of the SCN to external light–dark cycles [146]. Output signals from the SCN are in majority derived from the rhythmic changes in the efferent neuronal firing of the multiple neuronal populations that compose this region [147]. SCN projections are in the majority, restricted to other areas within the hypothalamus, and thalamus [148]. Although brain regions other than the SCN have been shown to display circadian rhythmicity, when isolated, their rhythms gradually dampen, indicating a dependence on the input from the SCN [149]. Rather than imposing rhythmicity, the role of the SCN is to synchronize the other brain regions [150].

### Peripheral circadian rhythmicity

Like in the brain, peripheral tissue cells also have endogenous circadian oscillators [139]. Rhythms of clock genes and proteins were observed in different tissues across the body [151, 152]. Many biological functions are under circadian control, particularly in relation to metabolism, including xenobiotic detoxification [153], glucose [154], and lipid metabolism [131, 155].

For the circadian system to be effective, it must be able to receive inputs from the environment and generate appropriate time‐keeping and synchrony across many different tissues. The SCN synchronizes peripheral tissues mainly through the endocrine and autonomic nervous system, but also by generating circadian oscillations in body temperature and feeding behavior [139, 156].

#### Endocrine system

Glucocorticoids are an output of the HPA axis and have widespread effects throughout the body, being key to maintaining homeostasis and responses to the environment [157, 158]. Daily rhythms in glucocorticoid release are one of the main cues from the brain to the periphery [159]. It is known that the timing of this rhythm is directly linked to the SCN, a discovery that actually helped identify the role of this nucleus in circadian control [137]. Administration of dexamethasone can lead to phase shift in the periphery, which indicates the role of glucocorticoids are an important entrainer of peripheral clocks [160]. This circadian role of glucocorticoids can be attributed to the fact that these hormones can regulate the expression of core clock genes [161]. Disruption or alterations to the rhythmic patterns of HPA axis activation and subsequent glucocorticoid release can impact the immune, metabolic, and neuronal function [162, 163, 164]. Additionally, recent evident also shows that corticosterone can modulate not only peripheral clock gene expression, but also in key brain regions, such as the hippocampus and cerebellum [165, 166].

Circulating levels of glucocorticoids are driven by CRF‐positive neurons residing in the PVN [167]. CRF neurons in the PVN display rhythmic clock gene expression that maintain daily rhythms in corticosterone [168]. The SCN receives light information and through neuronal projections to the PVN, relays important circadian information that entrain CRF neurons [52, 169, 170]. The importance of this is highlighted by the fact that light exposure at night can lead to altered rhythm of glucocorticoids, with loss of corticosterone rhythm and/or shifts in phase and amplitude [171, 172, 173]. As described previously, the activation of CRF neurons in the PVN leads to release of ACTH by the anterior pituitary and subsequent release of glucocorticoids by the adrenal cortex in circulation [52]. Rhythmic glucocorticoid release is maintained not only by central circadian signals, but also by a circadian clock within the adrenal gland cortex [174].

#### The autonomic nervous system

The ANS is one of the direct routes the SCN regulates peripheral clocks. In the liver, this has been demonstrated where removing innervation to the region led to a lack of clock gene response to light exposure [175]. Moreover, administering a GABAergic antagonist or glutamatergic antagonist to the PVN, an area that relays information from SCN to the liver in the form of sympathetic or parasympathetic activity, alters liver function which results in altered daily rhythms of plasma glucose [176]. One study utilizing salivary glands also demonstrated the importance of ANS inputs for circadian activity; here they indicated that, although it does not determine the phase of peripheral circadian oscillators, it has an important modulatory role [177]. Lastly, the ANS has been implicated in the modulation of timed glucocorticoid release by the adrenal gland, which indicated that light inputs in the SCN are communicated by sympathetic innervation to the adrenal cortex [178].

#### Other synchronizing cues: temperature and behavior

Light is the main circadian cue for the SCN, but peripheral clocks also use other environmental signals to keep track of time [179]. External temperature changes are weak entraining signals, since mammals are homeothermic animals, but changes in body temperature under the control of the SCN can act as a synchronizing cue to peripheral tissues [180]. Low‐amplitude changes in body temperature have been observed to alter circadian rhythmicity in fibroblasts, liver, pituitary, and lungs [181, 182].

Another important signal for peripheral clocks comes in the shape of feeding‐fasting cycles. Time of feeding is one of the main entraining cues for the liver clock and it can act independent of the SCN and the light cycle [183]. Moreover, when feeding time is restricted to the inactive phase, clock gene expression in the liver uncouples from clock gene expression in the SCN [184].

Lastly, social behavior can also act as a zeitgeber, modulating circadian rhythms [185]. Studies in mammals show that cohabitation can influence circadian rhythmicity [186, 187, 188]. The importance of the relationship between social interactions and the circadian rhythm becomes evident in mental health. Disruption of social rhythms is often observed in mood disorders [189], and social rhythm therapies have been proposed as a way to improve treatment responses and restore circadian biological processes [190].

### Gut microbiota and circadian interactions

Although a relatively new research area, there is growing evidence for interactions between the gut microbiota and the circadian rhythm, where microbial signals alter host circadian rhythmicity and vice versa [191, 192, 193]. More importantly, growing evidence points to circadian rhythms being an important factor to consider when studying the microbiota–gut–brain axis [194].

#### Rhythmic changes in microbiota composition

Like animals, bacteria also display circadian rhythmicity which have evolved as a response to the daily changes in selective pressures [195]. This was first shown in the mid‐1980s where research demonstrated that cyanobacteria displayed daily rhythms in nitrogen fixation and photosynthesis [196, 197, 198]. Although not as established, there is also evidence for endogenous circadian rhythms in nonphotosynthetic bacteria [199]. Circadian‐like rhythmicity was observed in the growth of *Klebsiella pneumonia* [200]. Moreover, evidence is also present for gut bacteria, where *Klebsiella aerogenes* (referred in the publication as *Enterobacter aerogenes*) displays endogenous circadian rhythmicity with a period close to 24 h that is entrained by melatonin [201].

Since the gut microbiota is a community, many studies also explored and demonstrated daily rhythms in microbial composition as a whole. A study in wild meerkats has found that the diurnal changes in bacterial load and composition of the gut microbiota are more pronounced than seasonal and lifetime dynamics [202]. In rodents, diurnal rhythmicity of gut microbes is well established [193, 203, 204, 205, 206, 207]. The average abundance of Bacteroidetes and Firmicutes, the two most abundant components of the mammalian microbiota oscillates during the day, with the peak of Bacteroidetes being around 11 PM and of Firmicutes around 7 AM [205]. Feeding time and nutrient availability are the most important synchronizing cues for the gut microbiota [206, 207]. Properties of the variation of the gut microbiota and its output across the day have been shown to be modulated by sex [205, 208, 209]. Additionally, diurnal oscillation of microbiota composition or individual members of the intestinal microbiota have also been observed in humans [210].

#### Gut microbiota modulation of host circadian rhythm

The daily activity of the host's feeding behavior changes and modulates microbiota composition, but rhythmicity of the microbiota can also affect the host's circadian rhythm. There is compelling evidence showing peripheral tissues responding to diurnal changes or modulation of the microbiota [20, 204]. These diurnal oscillations in microbial populations in the gut have been shown to shape the host's transcriptome in both liver and gut [193]. Moreover, the microbiota has also been shown to stabilize the gut's circadian rhythm increasing robustness to rapid changes in the environment [211]. Lastly, disruption of the gut microbiota by antibiotic or GF status have been reported to alter clock gene expression in the liver and the gut [203, 209, 212].

Microbial metabolites are responsible for many of the effects of the microbiota on the host through circulation. Unlike animals harboring a complex microbiota, the serum metabolome of GF and antibiotic‐treated mice does not display diurnal oscillations [193]. Oral administration of SCFAs have been shown to acutely change the phase of clock genes in the periphery [213]. Intraperitoneal injection of butyrate altered the Per2:Bmal1 mRNA ratio in the liver but not in the hypothalamus, indicating that peripheral clocks are more sensitive to microbial metabolites [204]. Additionally, the interplay between host and microbial tryptophan metabolism has been demonstrated to exhibit diurnal rhythmicity [203].

#### Circadian aspects of microbiota–host interactions

The microbiota can shape the host's health in different ways that include regulating not only the gut environment, but also the metabolic [214], immune [215], endocrine [216], and nervous systems [7]. Since these components of the host physiology and the gut microbiota display circadian rhythmicity, a growing body of research is exploring how circadian rhythms can impact microbiota–host interactions.

##### Metabolism

With both circadian rhythms and the microbiota being key modulators of metabolic activity, different works have explored how these two elements are integrated to shape metabolism. When administrated a high‐fat diet (HFD), animals develop metabolic syndrome and become obese; interestingly, germ‐free mice appear to be immune to these effects [204]. This is attributed to the fact that HFD leads to a disruption in the normal oscillations in microbial composition, in turn that generates aberrant microbial signals leading to rhythmic dysregulation of metabolic function [204]. Moreover, the body weight gain associated with HFD can be attenuated upon introduction of time‐restricted feeding during the active phase [217]. Lastly, the microbiota has also been reported to promote the diurnal levels of histone deacetylase 3 (HDAC3) in the intestinal epithelium, the rhythms in HDAC3 regulate histone acetylation, and furthermore, the diurnal oscillation in the expression of genes involved in metabolic processes [218].

##### Immune system

The majority of immune cell populations display expression of clock genes [219] and this endogenous clock regulates the inflammatory immune response [220]. In particular, type 3 innate lymphoid cells (ILC3s), which are circadian‐rhythmic, reside in the gut and produce rhythmic levels of cytokines and antimicrobial peptides, being able to regulate microbiota composition [221]. ILC3 rhythmicity is entrained from both light‐dependent inputs from the SCN and feeding behavior, regulating not only immunity but also the gut barrier [192, 222]. These rhythmic processes in innate immunity involving ILC3s are also regulated by the microbiota through the circadian clock [223]. Moreover, Toll‐like receptors (TLRs), a key component of the innate immune system, orchestrate the circadian communication between the host's gut tissue and the microbiota [20].

##### Endocrine system

As previously mentioned, endocrine signals are an important component of the circadian rhythm, regulating metabolism across the day and synchronizing peripheral tissues with the central clock [159]. Melatonin, an important hormone for regulating sleep/wake cycle and body temperature, displays diurnal rhythmicity [224]. This same hormone has been demonstrated to entrain rhythms in gut bacteria [201]. Glucocorticoids are a major synchronizing circadian cue that are known to be modulated by the microbiota [225]. Administration of dexamethasone, a synthetic glucocorticoid, leads to not only alterations in clock gene expression, but also changes in lipid metabolism and microbiota composition [226, 227].

##### Nervous system

Although there is compelling evidence demonstrating the importance of circadian rhythms in host–microbiota interactions, research has focused on the periphery and the effects on metabolism, with the consequences for the microbiota–gut–brain axis still largely unexplored [194]. Although this gap exists some of the work available gives insight about the importance of this relationship. Like the effects on body weight, time‐restricted feeding can also rescue some of the hippocampal impairments driven by HFD [228]. Moreover, antibiotic‐induced microbiota depletion leads to changes in the rhythmic metabolic profile in the brain, and more importantly, in the SCN [229]. Lastly, glucocorticoids are an important component of both circadian rhythm and gut–brain communication, indicating a role for microbiota to modulate circadian rhythmicity through the HPA axis [7, 23, 159].

## Integration of the stress response and circadian rhythm

Both the stress response and circadian rhythm are an evolutionarily conserved adaptation that allow an organism to respond to changes in the environment [230]. The importance of interplay between these two components in maintaining mental health can be observed in a disruption to clock‐regulated processes in stress‐related psychiatric disorders [231].

While the stress response is a rapid adaptive response to the unpredictable perceived danger [43], the circadian rhythm is an endogenous activated process that anticipates predictable environmental cues [132]. These two components share overlapping signaling pathways as the HPA axis and the ANS serve as distributors of both circadian and stress‐related information [18, 230]. Moreover, the brain areas that control the stress response, the PVN, and the master clock, the SCN, are close in proximity and interconnected [163, 168]. As the main output of the HPA axis, glucocorticoids work as major circadian and stress signaling molecules, synchronizing peripheral clocks with the SCN and acting as an effector of the stress response [232]. Although it has been known since the 1970s that time‐of‐day can impact the glucocorticoid stress response [47], the mechanisms underlying how the organism integrates circadian and stress inputs to maintain homeostasis are still being investigated.

### Circadian modulation of stress responsivity

Due to the circadian nature of glucocorticoids, animal studies indicated that when a stressor is conducted at the circadian peak of these hormones, the increase in circulating glucocorticoids is smaller than when the stressor is performed at the trough [47, 233, 234]. Additionally, *Bmal1* knockout mice display a dampened diurnal variation in glucocorticoids, that is coupled to an impaired stress response [235]. Similarly, mice that do not express *Per2* have also been reported to have an altered glucocorticoid rhythm [236], and *Per1* deficient mice display excessive grooming following acute stress and alterations in the expression of CRF in the PVN [237]. *Cry1/2* has been shown to modulate glucocorticoid responsivity [238]. Taken together, the available data on circadian clock and stress interactions indicates that clock genes can affect different aspects of the stress response leading to a modulation of stress responsivity across the day.

### Effects of stress on circadian rhythms

Just as daily rhythms of glucocorticoids and the clock machinery can alter stress responsivity, stress‐induced glucocorticoid release can lead to alterations in circadian rhythms [18]. Since glucocorticoids are one of the main synchronizing cues from the brain to the periphery, with rhythmic glucocorticoid release modulating clock gene expression [160], the effects of stress on the clock machinery are still being explored.

The adrenal clock, which regulates glucocorticoid secretion [174], displays changes in *Per2* phase following both chronic and acute stress [239]. Moreover, chronic mild stress‐induced changes in clock gene expression are also observed in the PFC and liver [240, 241]. Stressors, such as chronic social defeat or restraint, have also been shown to synchronize peripheral clocks, with the loss of synchrony induced by stress being dependent on the time‐of‐day of the stressor [242]. Since the SCN has been reported to not express glucocorticoid receptors [160], few studies report stress‐driven alterations in clock genes in this region. Early‐life stress has been shown to alter the expression of *Bmal1* in the SCN [243]. Similarly, in adulthood, repeated social defeat led to changes in gene expression in the SCN when the stressor was applied during the dark phase, but not the light [244].

### Gut microbiota as a regulator of both the stress response and circadian rhythm

**Fig. 4:**
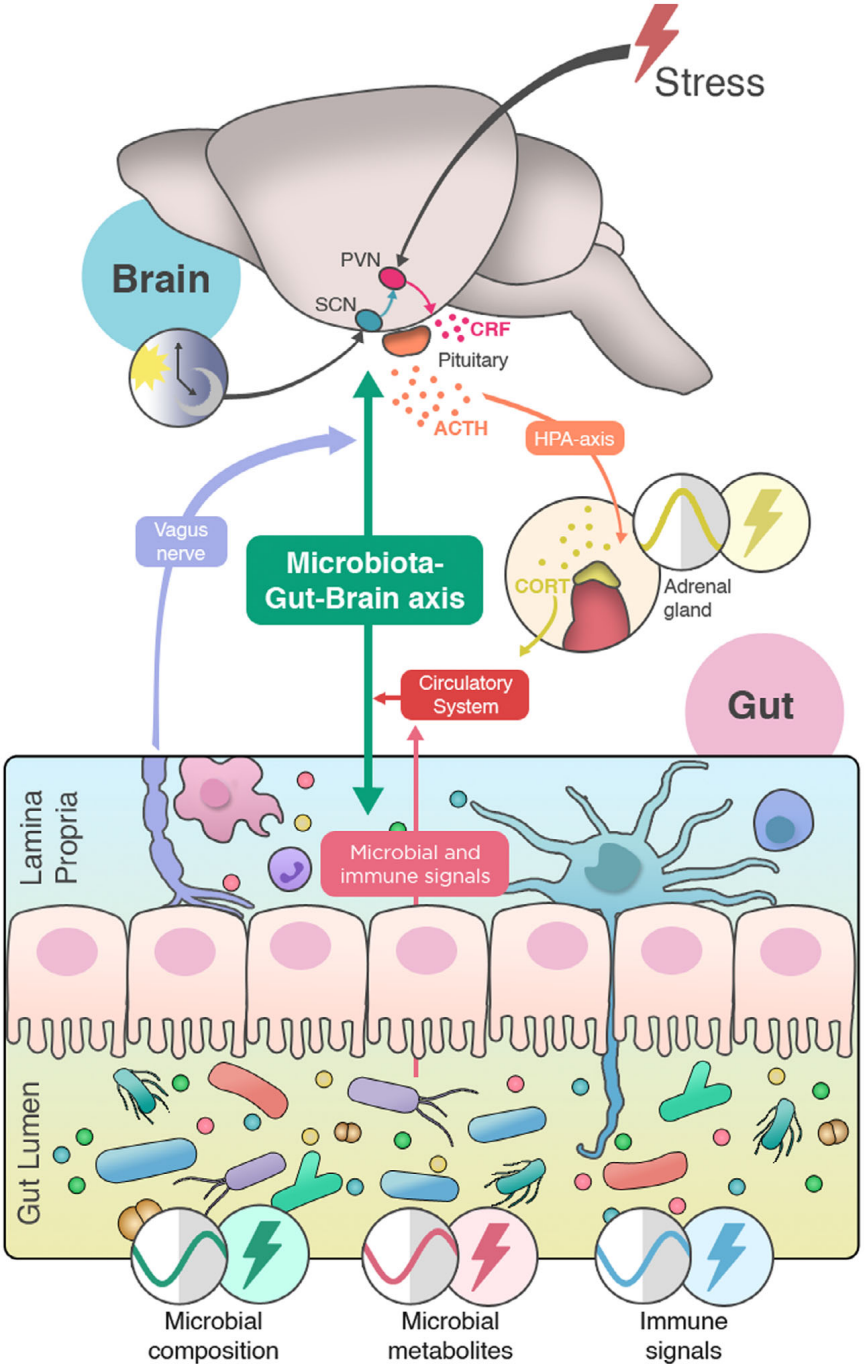
Interactions between stress, circadian rhythms, and microbiota–gut–brain axis. The HPA axis is at the intersection between stress, circadian rhythms, and the gut microbiota to brain communication. Glucocorticoids derived from HPA axis activation relay important circadian and stress information and are modulated by gut microbes. Moreover, the centers in the brain that regulate circadian and stress glucocorticoid release are in close proximity and interconnected.

New studies have been exploring the interplay between stress responsivity and circadian rhythm in the context of the microbiota–gut–brain axis (Fig. 4). Diurnal oscillations of microbes modulate the rhythms of glucocorticoids with implications to the stress response and behavior [245]. Microbial tryptophan metabolites are reported to be altered after stress and exhibit diurnal rhythmicity which are linked to changes in gut barrier function [203]. Sleep disruption has been shown to result in increased anxiety‐like behavior, and alter the microbiota composition and metabolome [246, 247]. Moreover, acetate has been shown to mitigate the effects of chronic sleep fragmentation on cognition [248], and administration of prebiotic diets improve sleep and circadian rhythmicity follwing sleep or circadian disruption [249, 250].

As previously described, it is well established that the gut microbiota can modulate circulating levels of glucocorticoids. HPA axis signaling is at the center of the stress response [158], circadian rhythms [159], and gut–brain communication [7]. Studies have explored how the gut microbiota can alter plasma glucocorticoids in the context of stress [16, 19], and at different times of the day [20], but the role of gut microbes in the modulation of diurnal rhythm of these hormones, and how this can lead to alterations in stress responsivity throughout the day, remains unknown in health and disease.

#### Circadian rhythm, stress, and microbiota–gut–brain axis dysfunction in disease

Evidence for a role of the gut microbiota in maintaining brain health has been growing [251]. Moreover, several of these conditions also present alterations to the stress response and circadian rhythm. The interaction between the microbiota–gut–brain axis, with stress and circadian rhythm becomes even more clear when we explore the literature of conditions that share a dysfunction in circadian and stress processes while presenting gut microbiota alterations.

##### Metabolic diseases

Metabolic diseases are maybe the best example of the interplay between the microbiota and circadian rhythm, with circadian disruption in both animal models and humans leading to changes in microbiota composition and increased susceptibility to metabolic syndrome [252, 253, 254, 255]. Besides changes in composition, microbial‐derived metabolites are also changed in shift workers and correlate with higher colon permeability [256]. Moreover, in the context of obesity, a study on a weight loss drug suggests an interaction with both the gut microbiota and circadian rhythms might underlie its beneficial effects [257]. Lastly, diurnal oscillations of the gut microbiota have also been implicated in metabolic disorders, with arrhythmic gut microbiota signatures shown to predict risk of developing type 2 diabetes [258].

The stress response also has been implicated in dysregulated metabolic conditions [259]. Stress exposure increases the risk of developing metabolic syndrome [260]. Chronic stress has been demonstrated to reduce the amplitude of glucocorticoid rhythm in rodents and humans [261, 262]. Flattening of diurnal glucocorticoid rhythms leads to altered glucose metabolism and fat accumulation [263]. Moreover, metabolic syndrome is characterized by alterations to microbiota composition [264].

##### Psychiatric disorders

Although there is a lack of studies investigating the interaction between circadian rhythms, stress, and the gut microbiota in the context of psychiatric and neurological disorders, studies have already investigated the role of these components independently. Circadian rhythm alterations are observed in many psychiatric conditions such as major depressive disorder (MDD), anxiety, schizophrenia, and bipolar disorder (BD) [127, 265, 266, 267, 268, 269]. Similar to metabolic syndrome, shift workers were also found to have a higher likelihood of developing adverse mental health outcomes, especially for depressive symptoms [270, 271, 272]. Moreover, the ‘extreme evening’ chronotype has also been found to correlate with higher levels of anxiety and depression [265]. In the case of BD, alterations to the circadian processes are observed as a disruption of social rhythms and sleep/awake cycles [273]. Additionally, a disruption of stress responsivity is also a hallmark of both MDD and BD, with individuals often exhibiting increased glucocorticoid levels and reduced sensitivity [274, 275]. Alterations in microbiota composition and function have also been observed in the same conditions [12, 276]. Interestingly, in animal models, prebiotics and probiotics have been demonstrated to have antidepressant‐like effects [30, 277].

Conditions such as alcohol use disorders (AUD) have also been implicated in altered circadian rhythms, stress, and microbiota. AUD is often associated with an evening chronotype and sleep disturbances [278, 279]. In parallel, stress is also associated with alcohol consumption, with stress exposure and HPA axis disfunction being an important factor for alcohol intake [280]. Moreover, circadian genes such as Per1 and Per2 have been demonstrated to play a role in regulating both baseline and stress‐induced alcohol consumption [281, 282]. Like the conditions previously mentioned, AUD also displays alterations to microbiota composition and other aspects of gut–brain communication [283].

Many psychiatric disorders often display altered sleep processes [284]. Stress is also known to impair sleep, while sleep deprivation is considered a stressor [285, 286, 287]. There has been growing evidence for the gut microbiota regulating sleep [288]. Evidence also suggests a possible role for gut microbes to modulate sleep in response to stress, with probiotics and microbial metabolite administration improving sleep upon stress exposure in both human and rodents [289, 290].

##### Neurodegenerative disorders

Like psychiatric disorders, many studies have explored the relationship between circadian rhythms and neurodegenerative disorders. Circadian dysfunction is a common symptom for individuals with Alzheimer's (AD), Parkinson's disease (PD), and Huntington's disease (HD) [291], and the motor and cognitive symptoms associated with these conditions display diurnal fluctuations [292]. Moreover, AD patients present with degeneration of the SCN [293], and PD patients display alterations in the normal rhythmicity of *Bmal1*, a gene important to maintain normal circadian rhythmicity [128, 294]. Like circadian rhythm, the stress response function is also playing an important role in the susceptibility, progression, and outcome of neurodegenerative disorders [295]. Perturbations in the gut microbial composition have also been observed in individuals with AD [296] and PD [297]. When it comes to PD, the gut microbiota has been shown to regulate motor deficits in an animal model, and FMT from PD patients to mice resulted in impaired motor performance [298]. Additionally, time‐restricted feeding has been reported to rescue changes in sleep, memory, and brain pathology in a mouse model of AD [299].

Taken together, the available literature on the conditions that display alterations in both circadian rhythm and stress responsivity are often accompanied by changes in gut microbiota composition. This indicates that the intricate relationship between these three components might play a role in regulating disease progression and symptoms. This exposes a gap in the current knowledge and demonstrates the need for more preclinical and clinical studies to understand how the gut microbiota and circadian rhythm can be targeted to improve quality of life of individuals that suffer from such conditions.

## Conclusions and future directions

With the modern environment involving increasing circadian disruption and stressor exposure, there is a need for a better understanding of how our responses to these constant changes are shaped, and more importantly, how they can be targeted to improve health. Although studying the interactions between the gut microbiota, stress, and circadian rhythms is an important research objective, some key questions remain to be addressed. More large‐scale longitudinal clinical studies need to be conducted in order to establish causality on the influence of the gut microbiota on stress responsivity and circadian rhythms [300]. Due to the challenging nature of circadian sampling in humans, use of innovative technologies will be key in providing high‐resolution data [301].

The translational aspects of findings in animal models to humans are also essential, as most of the work on circadian and microbiota interactions are so far being conducted in rodents. Although circadian oscillations of the gut microbiota are now well established [193, 203, 204, 205, 206, 207, 208, 211], most studies still do not report or account for time‐of‐day of sample collection, which might explain part of the variation and inconsistencies in microbiome datasets [302]. Understanding how the gut microbiota can shape circadian rhythms and stress responsivity through glucocorticoid release and other pathways can provide microbiota‐based tools that will be able to target stress and circadian manifestations at the same time for potential therapeutic benefit in a host of neuropsychiatric disorders.

## Conflict of interest

JFC has been an invited speaker at conferences organized by Bromotech and Nestle and has received research funding from Nutricia, Dupont/IFF, and Nestle. GC has received honoraria from Janssen, Probi, Apsen, and Ingelhem Boehringer as an invited speaker; is in receipt of research funding from Pharmavite, Fonterra, Reckitt, Nestle and Tate and Lyle; and has been paid for consultancy work by Yakult, Zentiva, Bayer Healthcare and Heel Pharmaceuticals. This support neither influenced nor constrained the contents of this preview. GSST declares no competing interests.

## Author contributions

GSST and JFC designed the review. GSST wrote the manuscript and designed the figures. JFC and GC reviewed and edited the manuscript.
